# Overcoming *Trypanosoma cruzi* persistence with a mechanistically distinct drug combination

**DOI:** 10.1038/s44259-026-00205-8

**Published:** 2026-04-30

**Authors:** Amanda Fortes Francisco, Francisco Olmo, Fanny Escudié, Eric Chatelain, John M. Kelly

**Affiliations:** 1 Department of Infection Biology, London School of Hygiene and Tropical Medicine, London, UK; 2 Department of Parasitology, Faculty of Sciences, University of Granada, Granada, Spain; 3 Drugs for Neglected Diseases initiative (DNDi), Geneva, Switzerland

**Keywords:** Diseases, Drug discovery, Microbiology

## Abstract

Infections with the protozoan parasite *Trypanosoma cruzi* are widespread in the Americas and can lead to severe cardiac and/or gastrointestinal pathology. Current treatments are limited to monotherapies characterised by prolonged dosing regimens, disputed efficacy and toxic side-effects. Sterile cure is often confounded by persistence of a small sub-population of parasites that display increased drug tolerance. Here, we demonstrate that short duration co-administration of well-tolerated sub-efficacious oral doses of the parasite-selective proteasome inhibitor GNF6702 and the pro-drug benznidazole produce parasitological cure in an experimental model of chronic Chagas disease.

## Introduction

Combination therapy has played a key role in controlling infectious diseases. With TB, malaria and HIV infection, for example, it has had a major impact by reducing treatment failures and minimising resistance^1–3^. However, this approach has yet to be applied to infections with the insect-transmitted hemoflagellate *Trypanosoma cruzi*. In the Americas, where more than 7 million people are affected, Chagas disease is the most serious parasitic infection^4,5^. In addition, due to migration it has become a global health challenge^6^. Infections with this obligate intracellular parasite are typically life-long and can lead to severe cardiac and gastrointestinal pathology, often with fatal outcomes^7,8^. The only approved treatments are benznidazole (BZ) or nifurtimox, nitroheterocyclic pro-drugs that share the same bioactivation mechanism^9^. In both cases, treatment is administered over prolonged periods (60–90 days), has variable efficacy, and is associated with adverse side-effects^10,11^, highlighting an urgent need for innovative therapeutic strategies.

**Fig. 1:**
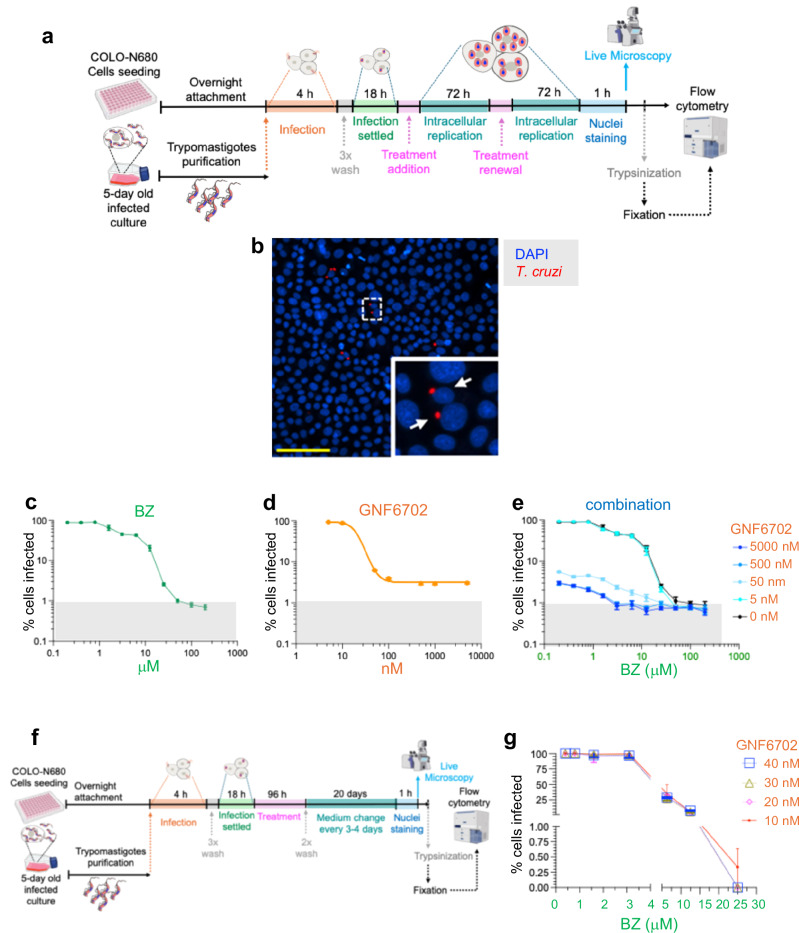
In vitro activity of GNF6702 and BZ against *T. cruzi.* **a** Protocol used to determine activity against intracellular amastigotes. **b** Fluorescence image of *T. cruzi* CL Brener PpyRE9h:mScarlet amastigotes (red) and COLO-N680 cell DNA (blue) following co-treatment with 6.3 μM BZ and 50 nM GNF6702, prior to flow cytometry. Yellow scale bar = 100 μM. Zoomed image shows cells containing single amastigotes. **c**–**e** Dose-response of BZ, GNF6702 and combination treatment, with the percentage of infected cells determined using flow cytometry (Methods). Grey area indicates infectivity <1%. Data are the mean ± SD (two independent biological replicates, three technical replicates). **f** Protocol of “wash-out” assay used to test combination treatment. **g** Dose-response of combination treatment, 20 days after drug removal, measured by flow cytometry.

**Fig. 2:**
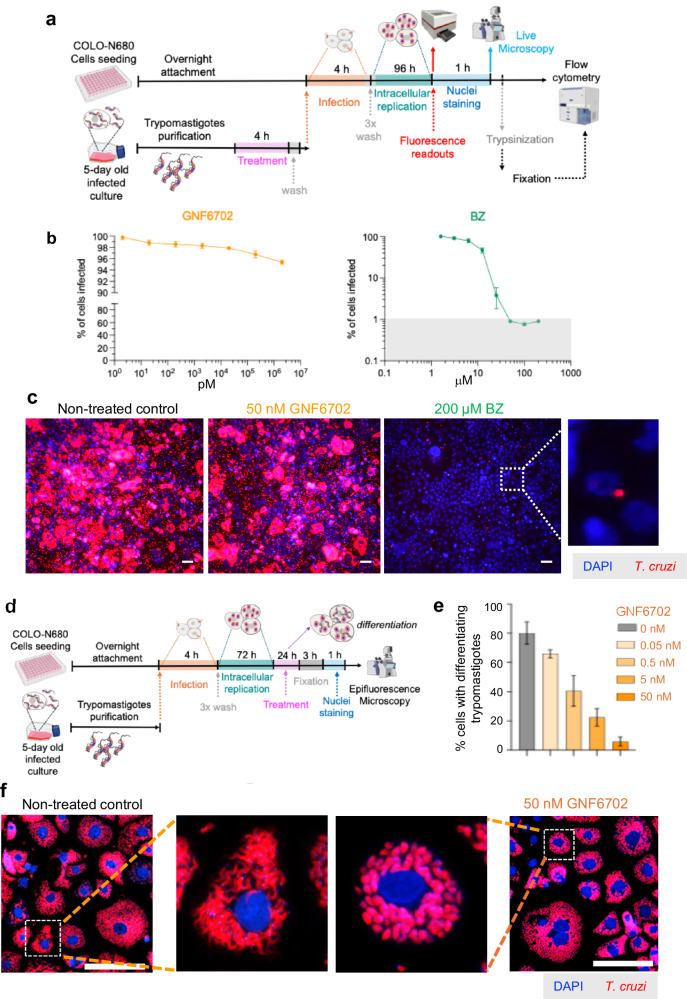
Impact of GNF6702 and BZ on *T. cruzi* trypomastigotes. **a** Schematic showing protocol used to assess the effect on trypomastigote infectivity. **b** Dose-response inhibition of COLO-N680 cell infection by *T. cruzi* trypomastigotes (CL Brener PpyRE9h:mScarlet strain) after 4 h treatment with GNF6702 and BZ. The percentage of cells infected was measured by flow cytometry (Methods). **c** Broad field images of infected COLO-N680 cells 96 h after invasion by drug-treated trypomastigotes (as above). Scale bars = 50 µm. **d** Protocol used to test the impact of GNF6702 on amastigote to trypomastigote differentiation. **e** Effect of GNF6702 on differentiation to trypomastigotes. Data (mean ± SD) were derived from 3 independent wells in which 60–80 infected cells were inspected. **f** Broad-field images of treated and non-treated cultures 96 h post-infection. Zoomed images show single infected cells. Scale bars = 100 µm.

**Fig. 3:**
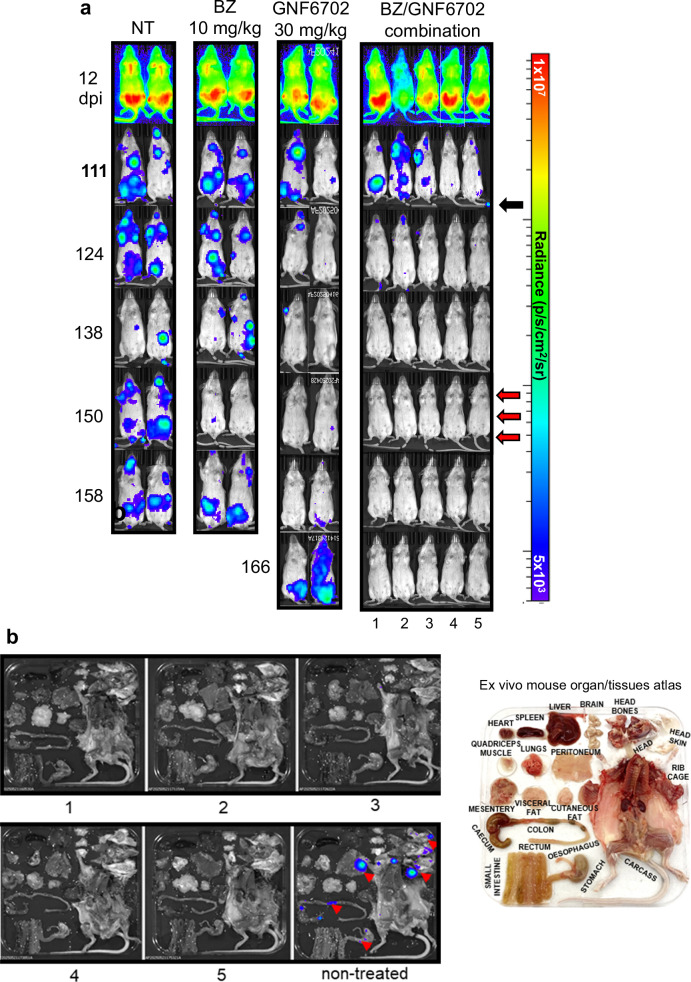
Efficacy of GNF6702:BZ combination therapy against chronic *T. cruzi* infection. **a** Ventral images of BALB/c mice infected with CL Brener PpyRE9h^21^, treated orally, once daily, for 10 days with 10 mg/kg benznidazole (BZ) and/or 30 mg/kg GNF6702 (Methods). Treatment was initiated 115 days post-infection (dpi) (black arrow). NT, non-treated. Mice were immunosuppressed using cyclophosphamide (3 × 200 mg/kg i.p.), administered at 3-day intervals (red arrows) starting 150 dpi. Heat-maps are on log10 scales and indicate bioluminescence intensity from low (blue) to high (red). **b** Ex vivo imaging of GNF6702:BZ treated mice (Methods). Organs and tissues were arranged as indicated in the atlas. Bioluminescent foci are highlighted by red arrows in non-treated image (right).

In *T. cruzi*, BZ undergoes reductive metabolism in a process initiated by the mitochondrial flavin-dependent nitroreductase TcNTR-1^9^. This leads to the production of a series of reactive intermediates^10,12^, giving rise to thiol-depletion and damage to DNA, lipids and proteins^13–17^. The small number of parasites that are able to survive treatment have been shown to be in a transient non-replicative state^17^, and host cells that remain infected typically contain only a single parasite. An ability to eliminate such persisters will be a requirement for any new Chagas disease therapeutics. GNF6702 is a triazolopyrimidine-based selective inhibitor that targets the proteasome in kinetoplastid protozoa^18^, and is well-tolerated in mice. The ubiquitin-proteasome system plays a pivotal role in protein homeostasis by degrading misfolded or damaged proteins and is essential for parasite survival and differentiation. GNF6702 exhibits a non-competitive mechanism of action and binds allosterically to the parasite proteasome, inhibiting its chymotrypsin-like activity. Oral treatment of mice with GNF6702 was reported to reduce *T. cruzi* levels in the blood, heart, and colon to undetectable levels^18^. However, as shown here (Figs. 1–3), GNF6702 monotherapy, both in vitro or in vivo, does not result in parasite elimination.

We hypothesised that combination therapy, based on a proteasome inhibitor (GNF6702) and a drug that can generate mis-folded/damaged proteins (BZ), might result in enhanced efficacy that is able to eliminate persister parasites. Using highly sensitive in vivo imaging, we demonstrate that short duration co-treatment with these drugs is able to cure infected mice at well-tolerated doses.

## Results

### Combination therapy in vitro is able to eliminate intracellular amastigotes

We first assessed the ability of monotherapy to eliminate intracellular amastigote forms of the parasite from COLO-N680 cells infected with the CL Brener PpyRE9h:mScarlet strain^19^ (Methods, Fig. 1a,b). With BZ alone, 6-day treatment at concentrations >50 µM was required to reduce the number of cells infected with *T. cruzi* to <1% (Fig. 1c). This is in line with previous studies^17^. When tested alone, GNF6702 concentrations greater than 25 nM led to a dramatic reduction in the number of infected cells, however complete parasite clearance could not be achieved, even at concentrations up to 5 μM. At all concentrations tested above 100 nM, the infection persisted in at least 3% of cells (Fig. 1d). These data indicate that under such conditions, a sub-population of parasites is refractory to GNF6702 over a wide range of doses. Combination treatment revealed potent activity and an ability to reduce or eliminate persister parasites. In the presence of 50 nM GNF6702, 3-4 times less BZ was required to reduce the number of infected cells to less than 1% (Fig. 1e). Furthermore, exposure to 30 nM GNF6702 and 25 µM BZ was sufficient to eliminate intracellular parasites, when assessed 20 days after drug removal (Fig. 1f, g; Supplementary Data Fig. 1). Thus, combining GNF6702 and BZ promotes parasite clearance.

### GNF6702 blocks differentiation of amastigotes into trypomastigotes

Intracellular amastigotes replicate by binary fission, and eventually differentiate into trypomastigotes, the flagellated, non-replicative, infectious form of *T. cruzi*. This is followed by host cell lysis (4–5 days post-infection in the case of the CL Brener strain) and parasite dissemination. Trypomastigotes often display reduced drug susceptibility compared to other life-cycle stages^20^. Here, we found that 4 h pre-treatment of trypomastigotes with GNF6702 at doses up to 2 μM had no significant effect on infectivity (Fig. 2a–c). To investigate the effect on differentiation of amastigotes to trypomastigotes, cultures of infected cells were treated for 24 h late in the infection cycle (72 h) (Fig. 2d). This revealed concentration-dependent arrested transition of differentiation into trypomastigotes (Fig. 2e, f), identifying a potential second means by which GNF6702 could act to enhance BZ potency. Amastigotes are the stage of the life-cycle most susceptible to BZ, whereas trypomastigotes are the least susceptible^20^. GNF6702-mediated proteasome inhibition^18^ therefore blocks differentiation into trypomastigotes, a life-cycle stage where greater BZ exposure would be required to eliminate the parasite.

### Combination therapy cures chronically infected mice

In vivo efficacy of BZ/GNF6702 combination therapy was assessed using highly sensitive bioluminescence imaging (BLI) of infected BALB/c mice^21^, a model that is widely used for Chagas disease drug assessment^22,23^. As predicted from in vitro analysis (Fig. 1d), GNF6702 monotherapy (10-day oral dosing) failed to cure chronically infected mice (Fig. 3a; Table 1), despite an initial knockdown in parasite burden. A similar outcome was observed during acute stage infections, even when treatment was prolonged for 20 days (Supplementary Data Fig. 2a). With BZ monotherapy, dosing at 30 mg/kg resulted in a partial cure rate of chronic infections (10-day treatment), but lower doses were ineffective (Table 1; Supplementary Data Fig. 2b). To assess combination therapy, we first treated mice with GNF6702 at 10 mg/kg (10-days, twice daily), in combination with 30 mg/kg BZ (once daily). This produced a partial cure rate (3/5). Next, we fixed GNF6702 at 30 mg/kg (once daily) and varied the BZ dose. At 10 mg/kg BZ and above, no parasites were detected in any mouse (*n* = 20) following 10-day treatment (Fig. 3a, b; Table 1). Curative outcomes were confirmed by post-treatment immunosuppression and ex vivo imaging of tissues and organs^24^ (Fig. 3b; Supplementary Data Fig. 2). All combination treatment regimens tested were well-tolerated.

**Table 1:** Cure rates in mice chronically infected with *T. cruzi* using different treatment regimens

Compound	Dose (10 days treatment)	Cure rate
BZ	3 mg/kg QD	0/5 (1)
BZ	10 mg/kg QD	0/10 (2)
BZ	20 mg/kg QD	0/5 (1)
BZ	30 mg/kg QD	7/12 (4)
GNF6702	10 mg/kg BID	0/5 (1)
GNF6702	30 mg/kg QD	0/14 (3)
BZ + GNF6702	3 mg/kg QD + 30 mg/kg QD	0/9 (1)
BZ + GNF6702	10 mg/kg QD + 30 mg/kg QD	**10/10** (2)
BZ + GNF6702	20 mg/kg QD + 30 mg/kg QD	**5/5** (1)
BZ + GNF6702	30 mg/kg QD + 30 mg/kg QD	**5/5** (1)
BZ + GNF6702	30 mg/kg QD + 10 mg/kg BID	3/5 (1)

Figures in brackets correspond to the number of independent experiments from which data were derived.

*QD* *quaque die* (once daily), *BID* *bis in die* (twice daily).

100% cure rates are highlighted in bold.

**Fig. 4:**
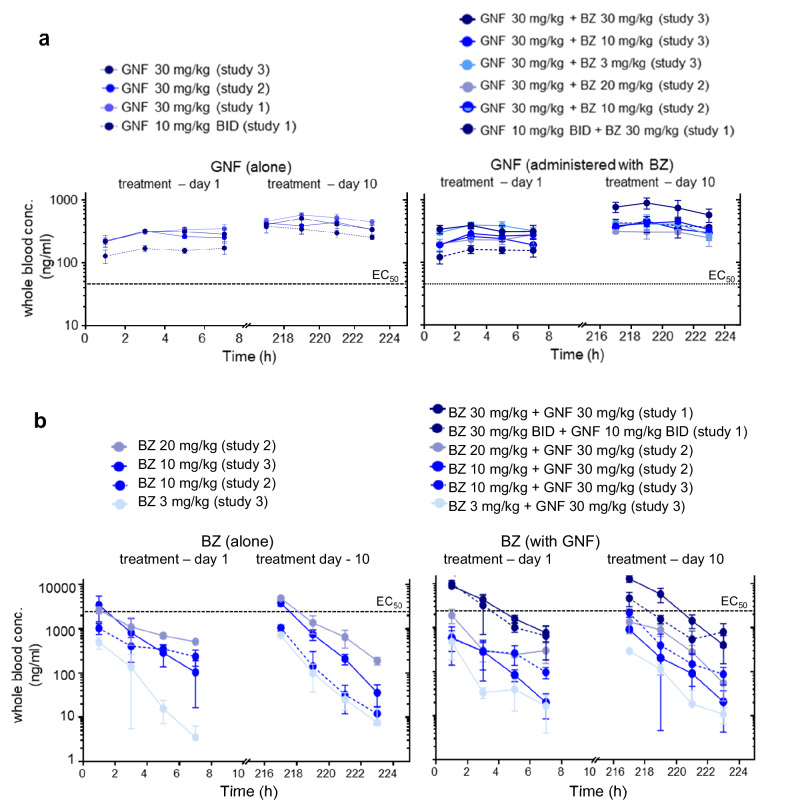
Pharmacokinetic profile of BZ and GNF6702 in mice following co-administration. Blood samples were taken from the tail vein of infected BALB/c mice on days 1 and 10 of treatment with BZ and GNF6702 alone, or in combination. Whole blood concentrations were quantified using LC-MS/MS (Methods). **a** Pharmacokinetic profile of GNF6702 alone, or co-administered with BZ. **b** Pharmacokinetic profile of BZ alone, or co-administered with GNF6702. Lines identify corresponding EC_50_ values.

The failure of GNF6702 monotherapy to eliminate parasites under the conditions tested (Fig. 3; Table 1) was not due to insufficient exposure levels. Plasma concentrations exceeded the free EC_90_ of this compound (fraction unbound in plasma = 0.063) across all treatment regimens tested, with slight accumulation at day 10 compared with day 1 (Fig. 4a). Thus, sustained exposure above the EC_90_ (30 mg/kg QD or 10 mg/kg BID) is not sufficient to achieve parasite elimination. In contrast, when combined with sub-efficacious BZ doses, full parasite clearance can be achieved. Blood concentrations for both drugs (Fig. 4a, b) were consistent with previously reported values^18,25^. Importantly, GNF6702/BZ co-administration did not alter exposure of either compound. Collectively, these findings imply that parasitological cure, mediated by combination therapy, results from the complementary modes of action of BZ and GNF6702 rather than from pharmacokinetic interactions.

## Discussion

Providing improved curative treatments for the large number of individuals in the asymptomatic chronic stage of *T. cruzi* infection is the major focus of the global Chagas disease drug development effort. The aim is to prevent progression to the symptomatic stage of the infection, where the resulting cardiac and/or gastrointestinal pathology is largely irreversible, and often fatal. Treatment failure with the front-line drug BZ is commonly reported^26–28^, even in patients who are PCR-ve in the immediate follow-up period. In mice, relapse of *T. cruzi* infection after BZ treatment is associated with the persistence in tissues of a small number of parasites that exist in a non-replicative state^17^. Once drug exposure is removed, these parasites can re-enter the cell-cycle and commence replication and dissemination. An essential property required of new Chagas disease drugs or therapeutic combinations is that they have an ability to eliminate these persisters. In the current study, we demonstrate that 10-day oral co-administration of the proteasome inhibitor GNF6702 (30 mg/kg) and low doses of BZ (10 mg/kg) produces a 100% cure rate in a well-validated model of Chagas disease, with no adverse side-effects. Combination therapy with these drugs, for which distinct modes of action have been established^9,12,18^, could therefore represent a promising option for treatment of this major Neglected Disease, for which there have been no newly approved therapeutics for more than 50 years.

With BZ monotherapy, curative outcomes were observed in some mice, although only at doses well above those that were successful in combination treatment (Table 1). In humans, the long duration period and doses currently used against *T. cruzi* infection can produce a range of toxic side effects, which often impact patient compliance^29^. Our data suggest there is potential, with combination therapy, for these parameters to be reduced considerably, even though GNF6702 on its own is ineffective at achieving sterile clearance (Figs. 1d, 3a). It can be inferred from our results that mechanistic features of BZ and GNF6702 activity interact to eliminate the small number of persister parasites that frequently confound sterile clearance. In *T. cruzi*, the reductive metabolism of BZ results in the generation of a range of reactive intermediates^10,12^. These can damage proteins directly or lead to increased mis-folding of newly synthesised proteins as a result of thiol depletion. One possibility is that accumulation of non-functional proteins, in a context of proteasome inhibition, may perturb *T. cruzi* cellular activity to an extent that is sufficient to kill the sub-population of parasites that would survive BZ monotherapy. Whatever the precise mechanism, our study demonstrates that combination treatment, using drugs with complementary modes of action, may be the way ahead for the curative treatment of an infection where novel therapeutic approaches are urgently needed.

## Methods

### Parasite/cell culture

Bioluminescent-fluorescent *T. cruzi* parasites (CL Brener PpyRE9h:mScarlet strain) and COLO-N680 cells (human oesophageal squamous cell carcinoma) were cultured in supplemented RPMI-1640 and Minimum Essential Medium (MEM, Sigma) respectively, as described^21,24^. For infections, tissue culture trypomastigotes (TCTs) were derived from infected cells and exposed to cell monolayers for 4 h. Extracellular parasites were then removed by washing with PBS, and flasks incubated with fresh medium for a further 5–7 days. Extracellular trypomastigotes were isolated by centrifugation of culture medium (1600 × *g*). Pellets were re-suspended in high-glucose (4.5 g/L) DMEM with 5% FBS and maintained at 37 °C until use. Motile trypomastigotes were counted using a haemocytometer.

### In vitro activity against *T. cruzi*

BZ and GNF6702 in vitro potency was determined by generating multiple-point potency curves by serial dilution in the corresponding culture medium. For amastigote assays, COLO-N680 cells in 100 µL growth medium were added to black, clear-bottomed, 96-well polystyrene microplates at 2.5 × 10^4^ cells/well. After overnight incubation, cells were infected with 5 × 10^5^ TCTs/well, a multiplicity of infection (MOI) of 10. Wells were then washed with PBS to remove non-internalized trypomastigotes, before adding 100 µL MEM supplemented with 5% FBS. Infections were allowed to establish overnight, then 100 µL MEM containing different drug concentrations was added. For assessment of activity against amastigote replication, 72-96 h post-incubation, plates were washed with PBS and stained with 2 µg/mL of Hoechst for 1 h. Cells were imaged by real-time epifluorescence microscopy using a Nikon Eclipse T2i. Cells were then washed with PBS and detached using TrypLE™ Express Enzyme at 37 °C, and fixed with 4% paraformaldehyde for 30 min. Cells were then washed in PBS, resuspended in flow cytometry staining buffer (PBS + 1% albumin) and fractionated in an Attune NxT Flow Cytometer. Gating was performed using a non-infected culture as a control. Activity at each concentration was normalized against values obtained from untreated infected cells. Non-infected, non-treated cells were used as negative controls to establish the detection threshold. For trypomastigote assays, the protocol followed the same principle with modifications as indicated (Fig. 2a). Dose-response curves were fitted, and 95% confidence intervals calculated using a non-lineal regression (curve fit) model and the equation [inhibitor] vs response variable slope applied in Graph Pad Prism 10 software (www.graphpad.com). All experiments were performed twice, independently, with three technical replicates per concentration, unless otherwise stated.

### Mice and parasites

Animal infections were performed under UK Home Office project license PPL P9AEE04E4/PP7589959 and approved by the LSHTM Animal Welfare and Ethical Review Board. All protocols and procedures were conducted in accordance with the UK Animals (Scientific Procedures) Act 1986 and with ARRIVE guidelines (https://arriveguidelines.org). Female BALB/c and CB17 SCID mice were purchased from Charles River (UK). Animals were maintained under specific pathogen-free conditions in individually ventilated cages. They experienced a 12 h light/dark cycle, with access to food and water ad libitum. SCID mice were infected with 1 × 10^4^ TCTs in 0.2 mL D-PBS via i.p. injection. Female BALB/c mice, aged 7–8 weeks, were infected by i.p injection with 1 × 10^3^ blood trypomastigotes derived from a SCID mouse^21,24^.

### Compounds and treatment

GNF6702 was synthesised by TCG, India and BZ by Epichem Pty Ltd., Australia. For in vitro assays, compounds were dissolved in DMSO (stock solutions of 8 mM for GNF6702 and 50 mM for BZ), aliquoted and stored at −20 °C until required. For the in vivo studies, BZ and GNF6702 were formulated at different concentrations in 0.5% (w/v) hydroxypropyl methylcellulose (HPMC) and 0.4% (v/v) Tween 80 in Milli-Q H_2_O and 0.5% methylcellulose, respectively. Drugs and vehicle were administered by oral gavage according to weights at the beginning of treatment. For all studies, vehicle treated mice were used as the negative control, and BZ as the positive control.

### In vivo and ex vivo bioluminescence imaging

For in vivo BLI, mice were injected with 150 mg/kg d-luciferin i.p., anaesthetized using 2.5% (v/v) isoflurane in oxygen for 2–3 min, and imaged using an IVIS Spectrum system (Revvity, MA, USA)^21,24^. Exposure times varied from 10 s to 5 min, depending on signal intensity. The detection threshold was established from uninfected mice. After imaging, mice were revived and returned to cages. For ex vivo imaging at the experimental end-points, mice were injected with 150 mg/kg d-luciferin i.p. 5 min before exsanguination under terminal anaesthesia using dolethal (200 mg/kg). Trans-cardiac perfusion was performed with 10 mL 0.3 mg/mL d-luciferin in DPBS (Dulbecco′s Phosphate Buffered Saline). Tissues and organs of interest were collected and arranged in squared Petri dishes, with the heart bisected along the coronal plane. Samples were soaked in DPBS containing 0.3 mg/mL d-luciferin. Bioluminescence imaging was performed as above. To estimate parasite burden, regions of interest were drawn using Living Image 4.8.2 to quantify bioluminescence expressed as total flux (photons/second)^21^.

### Assessment of BZ and GNF6702 exposure in blood

Following dosing on day 1 and 10, blood samples (10 μL) were taken from the tail vein of infected mice that had been treated with BZ or GNF6702 at 1, 3, 5 and 7 h, and transferred directly onto QIAcard FTA DMPK-B formats (QIAGEN; ref: WB129242) until analysis. Following extraction from the QIAcards, whole blood concentrations of BZ or GNF6702 were quantified using a validated LC-MS/MS method with a lower limit of quantification of 1.22 ng/mL. Calibration and QC samples met acceptance criteria per internal guidelines. Non-compartmental analysis was performed using Phoenix WinNonlin software, version 8.1. GNF6702 total to free exposure was corrected using a fraction unbound (Fu) in mouse plasma of 0.063 and 0.05 in assay medium, as described^30^. Similarly, a Fu of 0.68 in mouse plasma and 0.9 in assay medium was used for BZ.

### Statistical analyses

Data sets from the in vitro assays were obtained from the flow cytometer in an FCS file format. They were analysed using FlowJo 10.9.0 software and exported as Excel files. Raw data from the Excel document were pasted in GraphPad Prism Version 10.5.0. Groups were compared with ordinary one-way ANOVA with Tukey’s multiple comparisons test. A value of *P* < 0.5 was considered significant.

## Supplementary information

Supplementary Data

## Data Availability

All data analysed during this study are included in this published article and its supplementary information. Raw data are available on request from the corresponding authors.
