# Supplementary material for: Overcoming Trypanosoma cruzi persistence with a mechanistically distinct drug combination
Source: NPJ Antimicrob Resist. 2026 Apr 30;4:30. doi: 10.1038/s44259-026-00205-8 (PMC13133338; doi:10.1038/s44259-026-00205-8)
Supplement: Supplementary file 1 — Supplementary Data [file 44259_2026_205_MOESM1_ESM.pdf]

Supplementary Data: Francisco et al.

Supplementary Data Fig. 1 | In vitro assessment of GNF6702:BZ combination therapy against amastigotes in COLO-N680 cells. a, Broad field images illustrating the effect of GNF6702 treatment (6 days) on *T. cruzi* amastigote replication (CL Brener PpyRE9h:mScarlet strain). Scale bars=100 µm. **b**, Schematic of the combinational GNF6702:BZ "wash-out" assay. Each well was inspected exhaustively by epifluorescence microscopy to determine the presence of amastigotes, 20 days after drug removal. **c**, Broad field images of the wells in **b** at critical combination concentrations showing transition from "cure" to "non-cure" outcomes. Scale bars=50 µm.

Supplementary Data Fig. 2 | In vivo efficacy of GNF6702 and BZ in acute and chronic murine models of *T. cruzi* infection. BALB/c mice were infected with *T. cruzi* CL Brener PpyRE9h strain (21). Graphs show the total bioluminescence flux during infection and treatment of BALB/c mice (Methods) **a**, Acute stage monotherapy. **b**, Chronic stage monotherapy with BZ. The data relating to 30 mg/kg BZ were derived from one of four independent experiments. **c,** Chronic stage monotherapy with GNF6702. **d**, Chronic stage combination therapy. Grey and pink shading identifies the treatment and immunosuppression periods, respectively. The horizontal black line represents the background bioluminescence level, determined from non-infected mice.

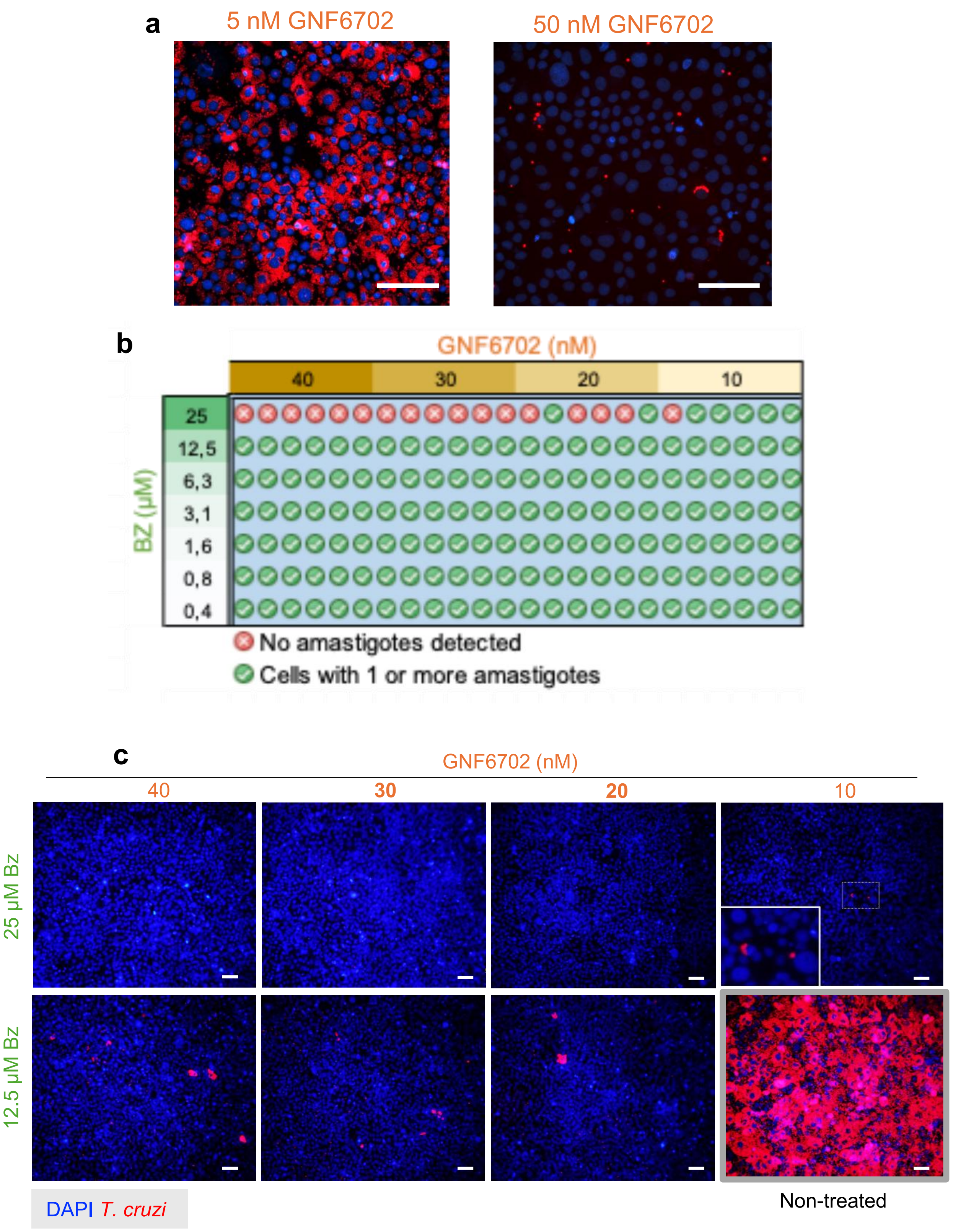

Supplementary Data Fig. 1

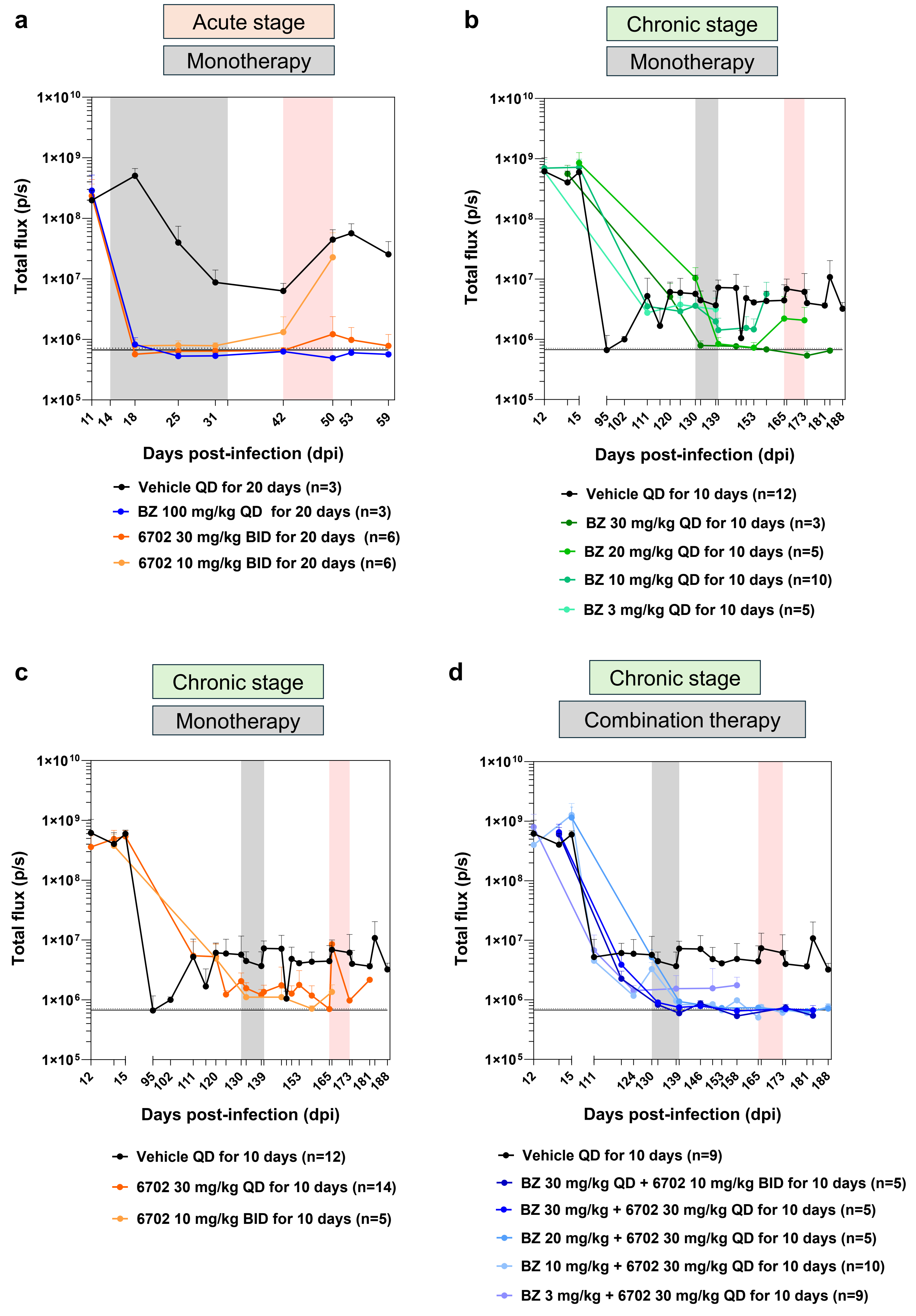

Supplementary Data Fig. 2
